# Supplementary material for: CRISPR/Cpf1-mediated DNA-free plant genome editing
Source: Nat Commun. 2017 Feb 16;8:14406. doi: 10.1038/ncomms14406 (PMC5316869; doi:10.1038/ncomms14406)
Supplement: Supplementary Information — Supplementary Figures, Supplementary Tables and Supplementary Note [file ncomms14406-s1.pdf]

Supplementary Information

Supplementary Figure 1

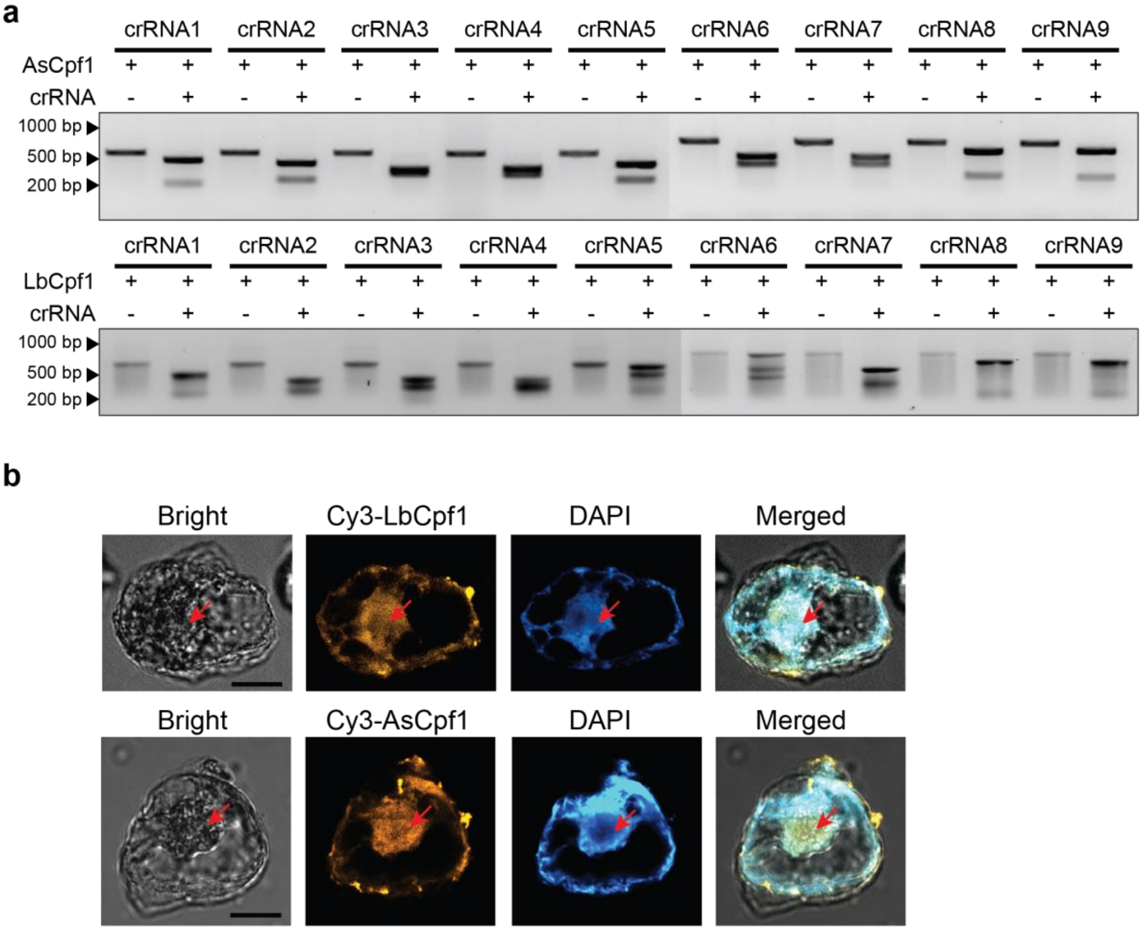

Supplementary Figure 1. *In vitro* cleavage assay for 9 crRNA-LbCpf1 complexes and localization of Cpf1 proteins in soybean protoplasts. (a) Preassembled AsCpf1-crRNA and LbCpf1-crRNA complexes cleave their target DNA *in vitro*. (b) Both Cy3-conjugated Cpf1 proteins were targeted to the nucleus of soybean protoplasts. The red arrow indicates the nucleus. DAPI, a nuclear marker. Bars = 10 μm.

Supplementary Figure 2

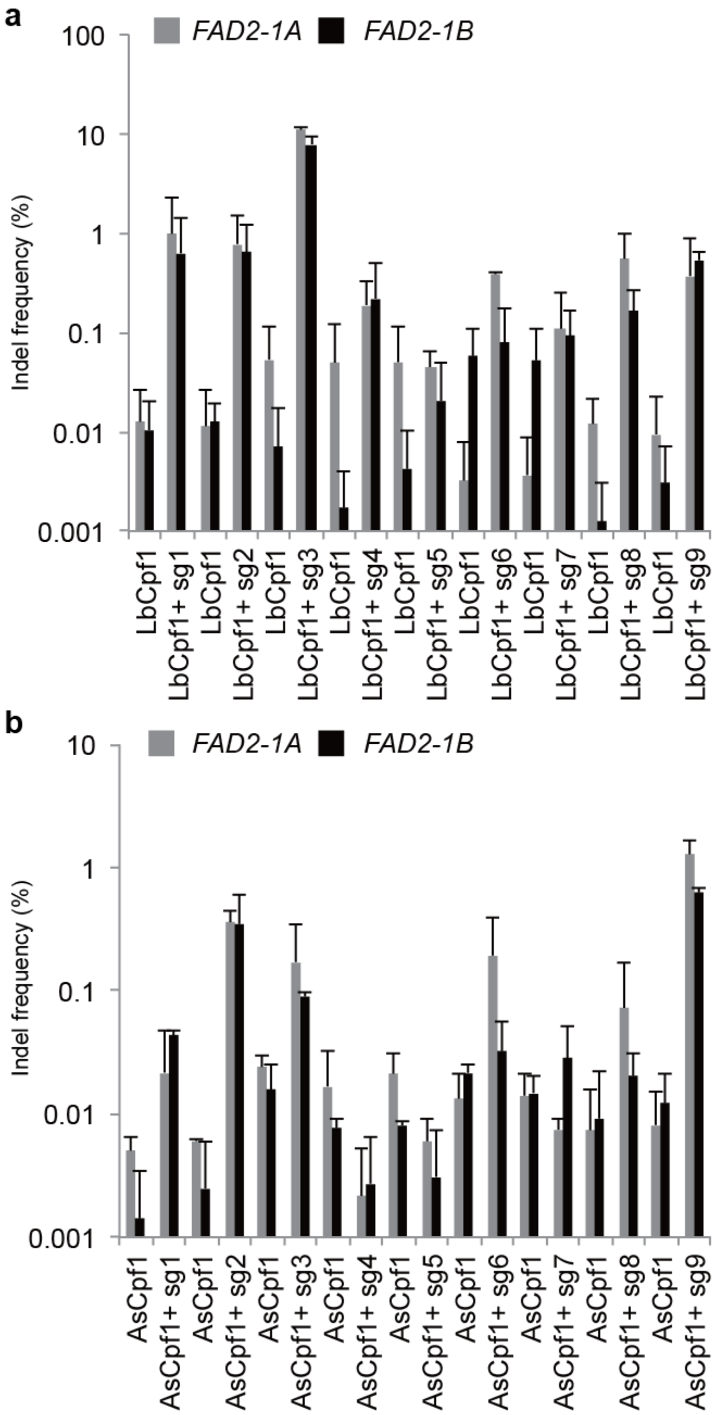

Supplementary Figure 2. Indel frequencies (%, Log10 scale at Y-axis) for 9 crRNAs at target sites in *FAD2-1A* and *FAD2-1B*. Error bars represent standard deviation (n = 2).

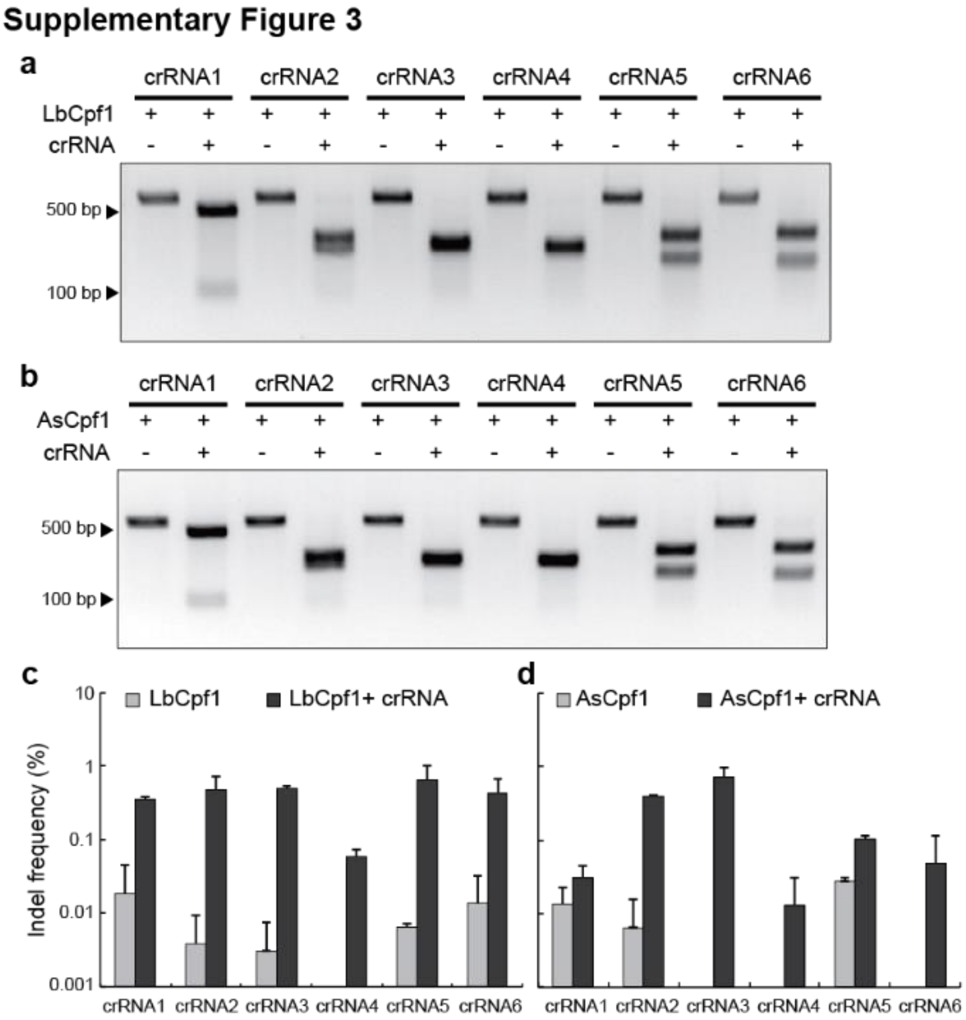

Supplementary Figure 3. CRISPR/Cpf1-mediated editing of the *AOC* gene in *Nicotiana attenuata* protoplasts. *In vitro* cleavage assay for 6 crRNAs complexed with (a) LbCpf1 and (b) AsCpf1 proteins. Indel frequencies (%, Log10 scale at Y-axis) at the target sites in protoplasts for 6 crRNAs complexed with (c) LbCpf1 and (d) AsCpf1 proteins. AOC, *ALLEN OXIDE CYCLASE*. Error bars represent standard deviation (n = 2).

Supplementary Figure 4

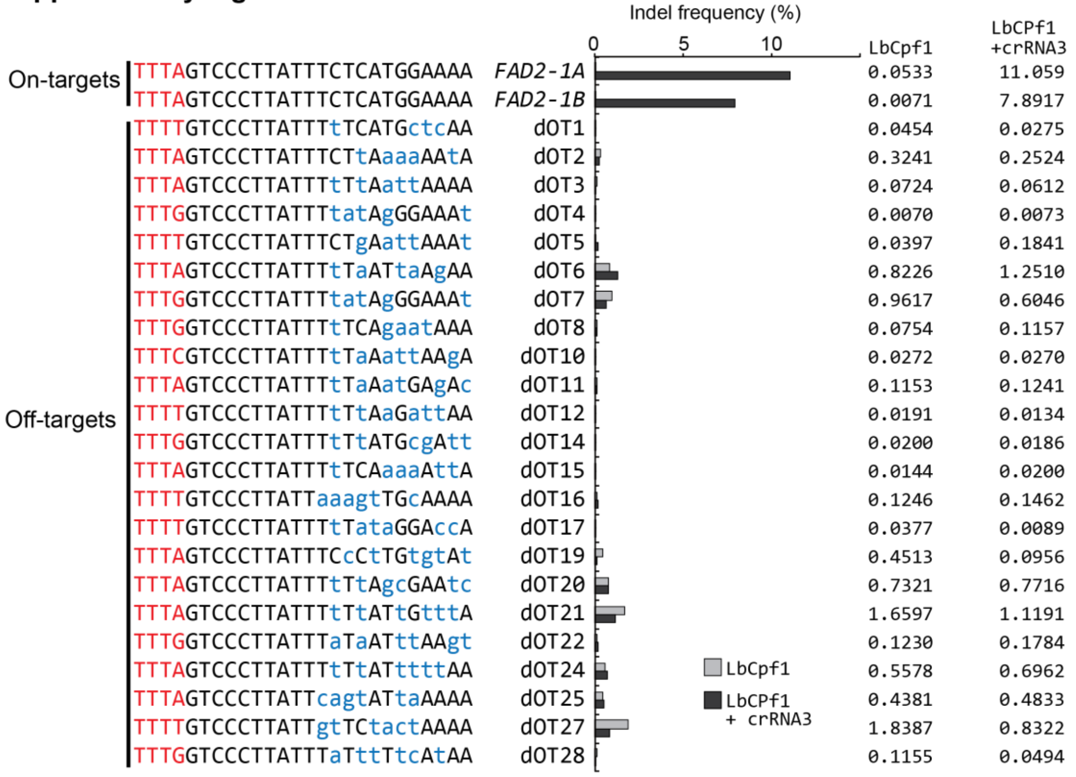

Supplementary Figure 4. *In vivo* evaluation of LbCpf1-crRNA3 activity at potential off-target sites in the genome. The indel frequencies (%) at 23 candidate distal off-target (dOT) sites (with up to 6 bp mismatches relative to the crRNAs) were measured and validated in LbCpf1-crRNA3-delivered soybean protoplasts by targeted deep sequencing. No mutations were detected at any of the 23 candidate loci. Red, PAM sequences; blue, mismatched nucleotide bases. Note, dOT21 and dOT27 have relatively high frequency of indel in some control samples, which we have frequently seen in AT-rich and A- or T-repeat regions[12]

Supplementary Figure 5

a

	Target *FAD2-1A*		Target *FAD2-1B*	
Transfected Plasmid	Total reads	Mutated reads (%)	Total reads	Mutated reads (%)
pLbCpf1+crRNA3	82087	37 (0.0 %)	87401	6 (0.0%)
pLbCpf1+crRNA9	87121	6 (0.0%)	78933	0 (0.0%)
pAsCpf1+crRNA3	77341	23 (0.0 %)	33586	4 (0.0%)
pAsCpf1+crRNA9	80967	66 (0.1%)	67301	18 (0.0%)

b

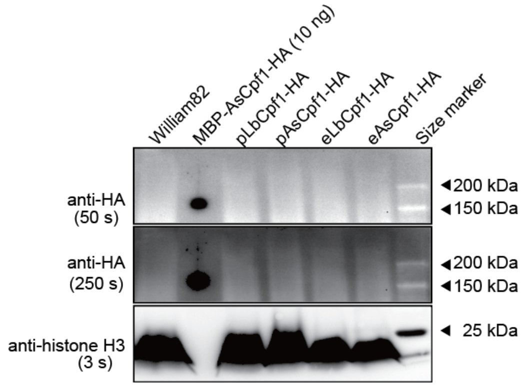

Supplementary Figure 5. Editing activity of plasmid-expressed Cpf1 and Cpf1 expression in soybean protoplasts. (a) Indel frequencies (%) at two FAD2 loci after transformation with the plasmid harboring plant-codon optimized LbCpf1-HA or AsCpf1-HA. (b) Protein expression from a plant codon (pLBCpf1/pAsCpf1) or *E. coli* codon (eLbCpf1/eAsCpf1) optimized Cpf1 plasmid in soybean protoplasts. The anti-HA antibody detects the Cpf1 protein and the anti-histone H3 antibody detects an endogenous soybean protein.

Supplementary Table 1. List of crRNAs used in this study.

Target gene	Name	[a]Sequence
Glycin max FAD2-1A/B	crRNA1	TTTCTACATTGCCACCACCTACTTCC
	crRNA2	TTTCCCTCATTGCATGGCCAATCTAT
	crRNA3	TTTAGTCCCTTATTTCTCATGGAAAA
	crRNA4	TTTCTCATGGAAAATAAGCCATCGCC
	crRNA5	TTTGTCCCAAAACCAAAATCCAAAGT
	crRNA6	TTTGGCTGCTATGTGTTTATGGGGTG
	crRNA7	TTTGGCAACTATGGACAGAGATTATG
	crRNA8	TTTGATGACACACCATTTTACAAGGC
	crRNA9	TTTACAAGGCACTGTGGAGAGAAGC
Nicotiana attenuata AOC	crRNA1	TTTGTTGCTAAAGGGGACAAGATCTCC
	crRNA2	TTTGATCAGAATGCAGAGTCCAGCCGT
	crRNA3	TTTCCTCTTCGTGTTTGATCAGAATGC
	crRNA4	TTTCTTTTCCTCTTCGTGTTTGATCAG
	crRNA5	TTTACAGCTTCTATTTCGGCGATTATG
	crRNA6	TTTCGGCGATTATGGTCACATCGCCGT

[a]Red indicates the PAM sequence.

Supplementary Table 2. List of putative off-target sequences of FAD2-targeted crRNA3 in the soybean genome.

[a]Off-target sequence	Chr.	Position	Direction	Mismatches	Name
TTTGGTCaCTaATTTCTCATGGAtcA	chr5	7021658	+	4	[b]OT1
TTTTGTCtCTTtTTTtTCATcGAAAA	chr6	14339586	+	4	OT2
TTTGGaCCCTTATTTacCATGGAcAA	chr7	9126069	-	4	OT3
TTTTGaCaCTcATTTtTCATGGAAAA	chr7	29877437	-	4	OT4
TTTTGTttCTTATTTCTCATtGAAAg	chr8	6892768	-	4	OT5
TTTCGTCCgTTATTTtTaATGaAAAA	chr8	20703738	-	4	OT6
TTTTGTCCCTTtTTTtTCATttAAAA	chr9	18027312	+	4	OT7
TTTTGTCaCaaATTTtTCATGGAAAA	chr10	2521140	+	4	OT8
TTTAGTCtCTTATTTCTaATaaAAAA	chr10	48025754	+	4	OT9
TTTGGTCCtTTATTTCcCATtGAtAA	chr11	28094966	+	4	OT10
TTTCtTCCCTTtTTTCTCATaaAAAA	chr12	20572847	-	4	OT11
TTTTGTCCCTTATTTtTCATGctcAA	chr17	2674940	+	4	OT12
TTTGGTCaCTaATTTCTCATGGAtcA	chr17	12553219	+	4	OT13
TTTTGTCCCTTATTTtTCATGctcAA	chr17	2674940	+	4	[c]dOT1
TTTAGTCCCTTATTTCTtAaaaAAtA	chr1	1189388	+	5	dOT2
TTTAGTCCCTTATTTtTtAattAAAA	chr9	3561633	-	5	dOT3
TTTGGTCCCTTATTTtatAgGGAAAt	chr11	15389742	+	5	dOT4
TTTTGTCCCTTATTTCTgAattAAAt	chr15	43359905	-	5	dOT5
TTTAGTCCCTTATTTtTaATtaAgAA	chr17	11701652	-	5	dOT6
TTTGGTCCCTTATTTtatAgGGAAAt	chr18	4987048	+	5	dOT7
TTTGGTCCCTTATTTtTCAgaatAAA	chr18	11727316	+	5	dOT8
TTTTGTCCCTTATTTCTgAattAAAt	chr18	44803139	-	5	dOT9
TTTCGTCCCTTATTTtTaAattAAgA	chr2	39113938	+	6	dOT10
TTTAGTCCCTTATTTtTaAatGAgAc	chr3	43408144	+	6	dOT11
TTTTGTCCCTTATTTtTtAaGattAA	chr3	43679365	+	6	dOT12
TTTAGTCCCTTATTTtTagcGGAttA	chr4	8732797	+	6	dOT13
TTTGGTCCCTTATTTtTtATGcgAtt	chr5	37988161	+	6	dOT14
TTTAGTCCCTTATTTtTCAaaaAttA	chr6	45888996	+	6	dOT15
TTTTGTCCCTTATTaaagtTGcAAAA	chr7	17897295	+	6	dOT16
TTTTGTCCCTTATTTtTataGGAccA	chr11	25098470	-	6	dOT17
TTTAGTCCCTTATTTtaaAaaaAAAA	chr13	40317930	-	6	dOT18
TTTAGTCCCTTATTTCcCtTGtgtAt	chr13	44326839	-	6	dOT19
TTTAGTCCCTTATTTtTtAgcGAAtc	chr14	45961325	-	6	dOT20
TTTAGTCCCTTATTTtTtATtGttta	chr15	9547205	-	6	dOT21
TTTGGTCCCTTATTTaTaATttAAgt	chr16	7599366	-	6	dOT22
TTTAGTCCCTTATTTtaaATatAAAc	chr16	29724887	+	6	dOT23
TTTAGTCCCTTATTTtTtATttttAA	chr17	38497391	+	6	dOT24
TTTAGTCCCTTATTcagtATtaAAAA	chr18	5202244	+	6	dOT25
TTTTGTCCCTTATTTCatATtaAttA	chr19	2704492	-	6	dOT26
TTTTGTCCCTTATTgtTCtactAAAA	chr19	45846421	+	6	dOT27
TTTGGTCCCTTATTTaTttTtcAtAA	chr19	49944362	-	6	dOT28
TTTTGTCCCTTATTTtaaATtGgAgA	chr20	26326125	+	6	dOT29

[a]Red indicates the mismatch sequence.

[b]OT indicates off-target sites with up to 4 nucleotide mismatches.

[c]dOT indicates off-target sites with up to 6 nucleotide mismatches in the distal region.

Supplementary Table 3. List of primers used for targeted deep sequencing.

Name	Sequence	Name	Sequence
FAD2-1A nested F	ATTGATAGCCCCTCCGTTCC	FAD2-1A nested R	AGTCATTACGCGGCAAATCC
FAD2-1A F1 crRNA1,2	CACCATTCACTGTTGGCCAA	FAD2-1A R1 crRNA1,2	CCCACAACATCATCAACCCA
FAD2-1A F2 crRNA3-5	CTGGTGTGTGGGTGATTGC	FAD2-1A R2 crRNA3-5	ACGAGAAGAGAAACAGCCCT
FAD2-1A F3 crRNA6,7	ACTCTCTCTACCGTGTTGCA	FAD2-1A R3 crRNA6,7	TGTAGAGAAGAGATGGTGAGCC
FAD2-1A F4 crRNA8,9	CAACCAATGCAATCAAGCCA	FAD2-1A R4 crRNA8,9	AGTCATTACGCGGCAAATCC
FAD2-1B nested F	GGCACCTACAATTATCAGCACT	FAD2-1B nested R	TGACAAAACTTCCATAACTCCCA
FAD2-1B F1 crRNA1,2	CACCATTCACTGTTGGCCAA	FAD2-1B R1 crRNA1,2	CCCATGGGTACTTGCTGAA
FAD2-1B F2 crRNA3-5	CCTTCAGCAAGTACCCATGG	FAD2-1B R2 crRNA3-5	CTGCCAGAGACATTGAAGGC
FAD2-1B F3 crRNA6,7	TTGCTCTACCGTGTTGCAAC	FAD2-1B R3 crRNA6,7	GCCTCCGTTGCATGGTAAT
FAD2-1B F4 crRNA8,9	ATAACTGATACTCATGTGGCTCA	FAD2-1B R4 crRNA8,9	TGACAAAACTTCCATAACTCCCA
FAD2-1A 2nd F1	ACACTCTTTCCCTACACGACGCTCTTCCGATCTCACCATTCACTGTTGGCCAA		
FAD2-1A 2nd R1	GTGACTGGAGTTCAGACGTGTGCTCTTCCGATCTCCCACAACATCATCAACCCA		
FAD2-1A 2nd F2	ACACTCTTTCCCTACACGACGCTCTTCCGATCTCTGGTGTGTGGGTGATTGC		
FAD2-1A 2nd R2	GTGACTGGAGTTCAGACGTGTGCTCTTCCGATCTACGAGAAGAGAAACAGCCCT		
FAD2-1A 2nd F3	ACACTCTTTCCCTACACGACGCTCTTCCGATCTACTCTCTCTACCGTGTTGCA		
FAD2-1A 2nd R3	GTGACTGGAGTTCAGACGTGTGCTCTTCCGATCTTGTAGAGAAGAGATGGTGAGCC		
FAD2-1A 2nd F4	ACACTCTTTCCCTACACGACGCTCTTCCGATCTCAACCAATGCAATCAAGCCA		
FAD2-1A 2nd R4	GTGACTGGAGTTCAGACGTGTGCTCTTCCGATCTAGTCATTACGCGGCAAATCC		
FAD2-1B 2nd F1	ACACTCTTTCCCTACACGACGCTCTTCCGATCTCACCATTCACTGTTGGCCAA		
FAD2-1B 2nd R1	GTGACTGGAGTTCAGACGTGTGCTCTTCCGATCTCCCATGGGTACTTGCTGAA		
FAD2-1B 2nd F2	ACACTCTTTCCCTACACGACGCTCTTCCGATCTCCTTCAGCAAGTACCCATGG		
FAD2-1B 2nd R2	GTGACTGGAGTTCAGACGTGTGCTCTTCCGATCTCTGCCAGAGACATTGAAGGC		
FAD2-1B 2nd F3	ACACTCTTTCCCTACACGACGCTCTTCCGATCTTTGCTCTACCGTGTTGCAAC		
FAD2-1B 2nd R3	GTGACTGGAGTTCAGACGTGTGCTCTTCCGATCTGCCTCCGTTGCATGGTAAT		
FAD2-1B 2nd F4	ACACTCTTTCCCTACACGACGCTCTTCCGATCTATAACTGATACTCATGTGGCTCA		
FAD2-1B 2nd R4	GTGACTGGAGTTCAGACGTGTGCTCTTCCGATCTTGACAAAACTTCCATAACTCCCA		
crRNA3 OT4MM-1 F	CCCAAAACTTTTTATGCTCGAAATTT	crRNA3 OT4MM-1 R	AAAACGTTCAATACACAACATTTAGGT
crRNA3 OT4MM-2 F	GCACGATGTGCGAAAGATT	crRNA3 OT4MM-2 R	TTGACCCTCCATTGTGTTGA
crRNA3 OT4MM-3 F	CCAACTTCAATTATGGGTGCTG	crRNA3 OT4MM-3 R	CGCATTATGCTTCATGTGACAA
crRNA3 OT4MM-4 F	ACACATGCGGATCCACAATTTTT	crRNA3 OT4MM-4 R	ATTGACATGCATGTATATATGTGTGT
crRNA3 OT4MM-5 F	CAACAGCGATGTTAAAATGTACAGATAG	crRNA3 OT4MM-5 R	CATGAGATTCAAAACTGCACCGT
crRNA3 OT4MM-6 F	AAATATGTAAAATGCTGACAAATTCATCTC	crRNA3 OT4MM-6 R	GGACATTCTTCCTGACGATAGC
crRNA3 OT4MM-7 F	TGACCATTTTGGAGTGACGACA	crRNA3 OT4MM-7 R	GACGAAAGACAGCGATTGGT
crRNA3 OT4MM-8 F	AGGAAATTTTAATCGACGGACCTTTTA	crRNA3 OT4MM-8 R	TCAAAAAATTTGGACGGAAAAATCCA
crRNA3 OT4MM-9 F	TAAGGATAGATTGAAAGTGATTAGTCA	crRNA3 OT4MM-9 R	GCAATTAGCATCGCTATCTAAAAAT
crRNA3 OT4MM-10 F	GCGCGTTTCATCTCTTTGAG	crRNA3 OT4MM-10 R	GCTGTATTAAAACTTAAAAGCATTCCC
crRNA3 OT4MM-11 F	CAAAGTGATGGCTTATAATGGAGAAC	crRNA3 OT4MM-11 R	GTTCCCTTCAGTGGCACAAC
crRNA3 OT4MM-12 F	GCGGTACGGTTCAGATAGCA	crRNA3 OT4MM-12 R	TCTACCGTAGAACTACACAGGTG
crRNA3 OT4MM-13 F	GGGCGCATTGTAAAGACTAAA	crRNA3 OT4MM-13 R	TTTGGTATCTTTTTCGATTTCTATCAG
LbCpf1crRNA	GAAATTAATACGACTCACTATAGGG AATTTCTACTAAGTGTAGAT		
LbCpf1 FAD2 sg1	GGAAGTAGGTGGTGGCAATGTAATCTACACTTAGTAGAAATT	LbCpf1 FAD2 sg2	ATAGATTGGCCATGCAATGAGGATCTACACTTAGTAGAAATT
LbCpf1 FAD2 sg3	TTTTCCATGAGAAATAAGGGACATCTACACTTAGTAGAAATT	LbCpf1 FAD2 sg4	GGCGATGGCTTATTTTCCATGAATCTACACTTAGTAGAAATT
LbCpf1 FAD2 sg5	ACTTTGGATTTTGGTTTTGGGAATCTACACTTAGTAGAAATT	LbCpf1 FAD2 sg6	CACCCCATAAACACATAGCAGCATCTACACTTAGTAGAAATT
LbCpf1 FAD2 sg7	CATAATCTCTGTCCATAGTTGCATCTACACTTAGTAGAAATT	LbCpf1 FAD2 sg8	GCCTTGTAAAATGGTGTGTCATATCTACACTTAGTAGAAATT
LbCpf1 FAD2 sg9	GCTTCTCTCCACAGTGCCTTGATCTACACTTAGTAGAAATT		
AsCpf1crRNA	GAAATTAATACGACTCACTATAGGG TAATTTCTACTCTTGTAGAT		
AsCpf1 FAD2 sg1	GGAAGTAGGTGGTGGCAATGTAATCTACAAGAGTAGAAATTA	AsCpf1 FAD2 sg2	ATAGATTGGCCATGCAATGAGGATCTACAAGAGTAGAAATTA
AsCpf1 FAD2 sg3	TTTTCCATGAGAAATAAGGGACATCTACAAGAGTAGAAATTA	AsCpf1 FAD2 sg4	GGCGATGGCTTATTTTCCATGAATCTACAAGAGTAGAAATTA
AsCpf1 FAD2 sg5	ACTTTGGATTTTGGTTTTGGGAATCTACAAGAGTAGAAATTA	AsCpf1 FAD2 sg6	CACCCCATAAACACATAGCAGCATCTACAAGAGTAGAAATTA
AsCpf1 FAD2 sg7	CATAATCTCTGTCCATAGTTGCATCTACAAGAGTAGAAATTA	AsCpf1 FAD2 sg8	GCCTTGTAAAATGGTGTGTCATATCTACAAGAGTAGAAATTA
AsCpf1 FAD2 sg9	GCTTCTCTCCACAGTGCCTTGATCTACAAGAGTAGAAATTA		

Supplementary Note 1. Sequence information of Cpf1-harboring vectors.

p2GW7-eAsCpf1-HA, *E. coli*-codon optimized AsCpf1.

Amp-R35s promoter 5’ NLS eAsCpf13’ NLS HA Stop codon35s terminator

GGCGTAATAGCGAAGAGGCCCGCACCGATCGCCCTTCCCAACAGTTGCGCAGCCTGAATGGCGAATGGACGCGCCCTGTAGCGGCGCATTAAGCGCGGCG
GGTGTGGTGGTTACGCGCAGCGTGACCGCTACACTTGCCAGCGCCCTAGCGCCCGCTCCTTTCGCTTTCTTCCCTTCCTTTCTCGCCACGTTCGCCGGCT
TTCCCCGTCAAGCTCTAAATCGGGGGCTCCCTTTAGGGTTCCGATTTAGTGCTTTACGGCACCTCGACCCCAAAAAACTTGATTAGGGTGATGGTTCACG
TAGTGGGCCATCGCCCTGATAGACGGTTTTTCGCCCTTTGACGTTGGAGTCCACGTTCTTTAATAGTGGACTCTTGTTCCAAACTGGAACAACACTCAAC
CCTATCTCGGTCTATTCTTTTGATTTATAAGGGATTTTGCCGATTTCGGCCTATTGGTTAAAAAATGAGCTGATTTAACAAAAATTTAACGCGAATTTTA
ACAAAATATTAACGCTTACAATTTCCTGATGCGGTATTTTCTCCTTACGCATCTGTGCGGTATTTCACACCGCATCAGGTGGCACTTTTCGGGGAAATGT
GCGCGGAACCCCTATTTGTTTATTTTTCTAAATACATTCAAATATGTATCCGCTCATGAGACAATAACCCTGATAAATGCTTCAATAATATTGAAAAAGG
AAGAGTATGAGTATTCAACATTTCCGTGTCGCCCTTATTCCCTTTTTTGCGGCATTTTGCCTTCCTGTTTTTGCTCACCCAGAAACGCTGGTGAAAGTAA
AAGATGCTGAAGATCAGTTGGGTGCACGAGTGGGTTACATCGAACTGGATCTCAACAGCGGTAAGATCCTTGAGAGTTTTCGCCCCGAAGAACGTTTTCC
AATGATGAGCACTTTTAAAGTTCTGCTATGTGGCGCGGTATTATCCCGTATTGACGCCGGGCAAGAGCAACTCGGTCGCCGCATACACTATTCTCAGAAT
GACTTGGTTGAGTACTCACCAGTCACAGAAAAGCATCTTACGGATGGCATGACAGTAAGAGAATTATGCAGTGCTGCCATAACCATGAGTGATAACACTG
CGGCCAACTTACTTCTGACAACGATCGGAGGACCGAAGGAGCTAACCGCTTTTTTGCACAACATGGGGGATCATGTAACTCGCCTTGATCGTTGGGAACC
GGAGCTGAATGAAGCCATACCAAACGACGAGCGTGACACCACGATGCCTGTAGCAATGGCAACAACGTTGCGCAAACTATTAACTGGCGAACTACTTACT
CTAGCTTCCCGGCAACAATTAATAGACTGGATGGAGGCGGATAAAGTTGCAGGACCACTTCTGCGCTCGGCCCTTCCGGCTGGCTGGTTTATTGCTGATA
AATCTGGAGCCGGTGAGCGTGGGTCTCGCGGTATCATTGCAGCACTGGGGCCAGATGGTAAGCCCTCCCGTATCGTAGTTATCTACACGACGGGGAGTCA
GGCAACTATGGATGAACGAAATAGACAGATCGCTGAGATAGGTGCCTCACTGATTAAGCATTGGTAACTGTCAGACCAAGTTTACTCATATATACTTTAG
ATTGATTTAAAACTTCATTTTTAATTTAAAAGGATCTAGGTGAAGATCCTTTTTGATAATCTCATGACCAAAATCCCTTAACGTGAGTTTTCGTTCCACT
GAGCGTCAGACCCCGTAGAAAAGATCAAAGGATCTTCTTGAGATCCTTTTTTTCTGCGCGTAATCTGCTGCTTGCAAACAAAAAAACCACCGCTACCAGC
GGTGGTTTGTTTGCCGGATCAAGAGCTACCAACTCTTTTTCCGAAGGTAACTGGCTTCAGCAGAGCGCAGATACCAAATACTGTTCTTCTAGTGTAGCCG
TAGTTAGGCCACCACTTCAAGAACTCTGTAGCACCGCCTACATACCTCGCTCTGCTAATCCTGTTACCAGTGGCTGCTGCCAGTGGCGATAAGTCGTGTC
TTACCGGGTTGGACTCAAGACGATAGTTACCGGATAAGGCGCAGCGGTCGGGCTGAACGGGGGGTTCGTGCACACAGCCCAGCTTGGAGCGAACGACCTA
CACCGAACTGAGATACCTACAGCGTGAGCTATGAGAAAGCGCCACGCTTCCCGAAGGGAGAAAGGCGGACAGGTATCCGGTAAGCGGCAGGGTCGGAACA
GGAGAGCGCACGAGGGAGCTTCCAGGGGGAAACGCCTGGTATCTTTATAGTCCTGTCGGGTTTCGCCACCTCTGACTTGAGCGTCGATTTTTGTGATGCT
CGTCAGGGGGGCGGAGCCTATGGAAAAACGCCAGCAACGCGGCCTTTTTACGGTTCCTGGCCTTTTGCTGGCCTTTTGCTCACATGTTCTTTCCTGCGTT
ATCCCCTGATTCTGTGGATAACCGTATTACCGCCTTTGAGTGAGCTGATACCGCTCGCCGCAGCCGAACGACCGAGCGCAGCGAGTCAGTGAGCGAGGAA
GCGGAAGAGCGCCCAATACGCAAACCGCCTCTCCCCGCGCGTTGGCCGATTCATTAATGCAGCTGGCACGACAGGTTTCCCGACTGGAAAGCGGGCAGTG
AGCGCAACGCAATTAATGTGAGTTAGCTCACTCATTAGGCACCCCAGGCTTTACACTTTATGCTTCCGGCTCGTATGTTGTGTGGAATTGTGAGCGGATA
ACAATTTCACACAGGAAACAGCTATGACCATGATTACGCCAAGCTATTTAGGTGACACTATAGAATACTCAAGCTATGCATCCAACGCGTTGGGAGCTCT
CCCATATGGTCGACTAGAGCCAAGCTGATCTCCTTTGCCCCGGAGATCACCATGGACGACTTTCTCTATCTCTACGATCTAGGAAGAAAGTTCGACGGAG
AAGGTGACGATACCATGTTCACCACCGATAATGAGAAGATTAGCCTCTTCAATTTCAGAAAGAATGCTGACCCACAGATGGTTAGAGAGGCCTACGCGGC
AGGTCTCATCAAGACGATCTACCCGAGTAATAATCTCCAGGAGATCAAATACCTTCCCAAGAAGGTTAAAGATGCAGTCAAAAGATTCAGGACTAACTGC
ATCAAGAACACAGAGAAAGATATATTTCTCAAGATCAGAAGTACTATTCCAGTATGGACGATTCAAGGCTTGCTTCATAAACCAAGGCAAGTAATAGAGA
TTGGAGTCTCTAAGAAAGTAGTTCCTACTGAATCAAAGGCCATGGAGTCAAAAATTCAGATCGAGGATCTAACAGAACTCGCCGTGAAGACTGGCGAACA
GTTCATACAGAGTCTTTTACGACTCAATGACAAGAAGAAAATCTTCGTCAACATGGTGGAGCACGACACTCTCGTCTACTCCAAGAATATCAAAGATACA
GTCTCAGAAGACCAAAGGGCTATTGAGACTTTTCAACAAAGGGTAATATCGGGAAACCTCCTCGGATTCCATTGCCCAGCTATCTGTCACTTCATCAAAA
GGACAGTAGAAAAGGAAGGTGGCACCTACAAATGCCATCATTGCGATAAAGGAAAGGCTATCGTTCAAGATGCCTCTGCCGACAGTGGTCCCAAAGATGG
ACCCCCACCCACGAGGAGCATCGTGGAAAAAGAAGACGTTCCAACCACGTCTTCAAAGCAAGTGGATTGATGTGATATCTCCACTGACGTAAGGGATGAC
GCACAATCCCACTATCCTTCGCAAGACCCTTCCTCTATATAAGGAAGTTCATTTCATTTGGAGAGGACTCCGGTATTTTTACAACAATACCACAACAAAA
CAAACAACAAACAACATTACAATTTACTATTCTAGTCGACCTGCAGGCGGCCGCACTAGTGATATCACAAGTTTGTACAAAAAAGCAGGCTTCATGGGTG
CTCCTCCAAAAAAGAAGAGAAAGGTAGGTGGTATGACACAGTTTGAAGGCTTCACCAATCTCTACCAGGTCAGCAAGACGCTACGTTTTGAGCTTATCCC
GCAGGGAAAAACCCTGAAACACATTCAGGAACAGGGGTTCATAGAGGAAGATAAGGCGCGTAACGACCATTATAAAGAACTGAAGCCTATAATCGACCGT
ATTTATAAAACGTACGCGGATCAGTGCCTGCAGCTGGTTCAGCTGGATTGGGAGAATCTGTCCGCGGCTATTGATAGCTATCGCAAAGAGAAGACCGAGG
AAACCCGTAACGCACTGATTGAAGAGCAGGCGACCTATCGGAATGCGATCCATGATTACTTCATCGGCCGCACCGACAACCTGACCGATGCAATTAACAA
ACGTCACGCAGAGATTTACAAAGGTCTGTTTAAAGCAGAGTTATTCAATGGCAAGGTTCTGAAACAGCTGGGTACGGTCACCACCACCGAACACGAAAAC
GCACTGCTGAGGAGCTTTGATAAATTTACCACATATTTCAGCGGTTTCTATGAAAATCGTAAGAATGTATTTAGCGCCGAAGATATTTCCACCGCAATTC
CTCATCGTATTGTGCAGGATAATTTTCCGAAGTTTAAAGAAAATTGTCATATTTTTACCCGTCTGATCACCGCGGTACCGAGCCTGCGAGAGCATTTTGA
AAACGTTAAGAAAGCCATTGGAATTTTTGTCAGTACCAGCATTGAAGAAGTGTTTTCGTTCCCGTTCTATAACCAACTGCTGACCCAGACCCAGATTGAT
CTGTACAATCAGCTGCTGGGGGGCATAAGCCGCGAGGCAGGTACCGAAAAGATAAAGGGACTCAATGAGGTGCTGAATCTGGCAATTCAGAAGAATGATG
AAACGGCTCATATCATTGCTAGCCTGCCGCATCGTTTCATTCCCCTGTTTAAGCAAATCCTGAGCGATCGCAATACACTGAGCTTTATCCTCGAAGAGTT
TAAATCGGACGAAGAAGTTATCCAGAGCTTTTGCAAATACAAAACCCTGCTGCGGAACGAAAATGTGCTGGAGACCGCTGAAGCACTGTTTAATGAACTG
AACTCGATCGACCTCACCCATATTTTTATATCCCACAAAAAACTGGAAACCATAAGCAGCGCTCTGTGTGACCATTGGGATACCCTGCGCAACGCCCTGT
ATGAACGGCGTATCAGCGAGCTGACCGGGAAAATCACCAAATCCGCAAAGGAAAAAGTTCAGCGTAGTCTGAAACACGAGGACATCAACCTGCAAGAAAT
TATTAGCGCAGCAGGTAAAGAGCTGAGCGAAGCATTCAAACAGAAAACCAGCGAAATCCTGAGCCATGCCCATGCTGCACTGGATCAGCCGCTGCCGACC
ACCCTGAAAAAACAGGAGGAAAAGGAGATTCTGAAAAGCCAACTGGACAGCCTGCTGGGCCTGTATCACCTGCTGGACTGGTTTGCAGTCGATGAGAGCA
ACGAGGTTGATCCTGAGTTCTCCGCTCGTCTGACCGGAATCAAGCTGGAGATGGAACCGAGTCTGTCGTTTTACAATAAAGCGCGTAATTACGCGACCAA
GAAACCGTATAGCGTGGAAAAATTCAAACTGAACTTTCAGATGCCGACCCTTGCAAGCGGATGGGACGTTAACAAAGAAAAAAACAATGGGGCAATTCTG
TTTGTGAAAAATGGCCTCTATTATCTGGGTATCATGCCGAAACAGAAAGGGCGCTACAAAGCCCTGTCATTTGAGCCGACCGAGAAAACCTCAGAGGGTT
TCGACAAGATGTACTACGATTATTTCCCGGATGCGGCAAAAATGATACCCAAATGTAGCACCCAACTGAAGGCAGTTACAGCCCACTTTCAGACCCATAC
CACCCCGATCCTGCTGTCGAACAATTTTATAGAGCCGCTGGAAATTACCAAAGAGATTTATGATCTGAATAATCCGGAAAAGGAGCCCAAGAAATTTCAG
ACGGCGTATGCAAAAAAGACCGGGGATCAGAAAGGTTATCGTGAAGCGCTGTGCAAATGGATTGACTTTACCCGTGACTTTCTGTCAAAATATACCAAAA
CGACGAGCATTGATCTGAGCAGCCTACGTCCGAGCAGCCAATATAAGGATCTGGGCGAATATTACGCCGAACTGAATCCGCTGCTCTACCATATTTCCTT
CCAACGAATCGCTGAAAAAGAAATAATGGACGCCGTTGAAACCGGCAAACTGTATCTGTTTCAAATCTACAACAAAGATTTCGCCAAAGGCCATCACGGT
AAGCCGAACCTGCATACCCTGTATTGGACCGGTCTGTTTAGCCCGGAGAATCTGGCCAAAACCAGCATCAAGCTGAACGGACAGGCAGAACTGTTTTACC
GCCCCAAAAGCCGTATGAAAAGGATGGCACACCGCCTGGGCGAAAAAATGCTGAATAAGAAACTCAAAGATCAGAAAACGCCGATACCGGATACCCTTTA
TCAGGAGCTGTATGATTATGTTAACCACCGGCTGAGCCATGACCTGAGCGACGAAGCGCGTGCACTGCTGCCGAACGTGATTACCAAGGAAGTCTCGCAT
GAAATTATTAAAGATCGGCGCTTCACCAGTGATAAATTTTTCTTCCATGTACCGATCACCCTGAATTATCAAGCCGCAAATAGCCCTTCCAAATTTAATC
AACGCGTGAATGCGTACCTGAAAGAGCATCCGGAGACCCCAATTATTGGCATAGACCGAGGAGAACGCAATCTCATTTATATCACCGTCATTGATAGCAC
CGGTAAGATCCTGGAACAGCGTAGCCTGAATACCATTCAGCAGTTTGACTACCAGAAAAAGCTGGACAACAGAGAAAAGGAACGTGTAGCCGCCCGGCAG

GCTTGGAGTGTGGTGGGTACTATCAAGGATCTGAAGCAGGGGTATCTCTCCCAAGTTATCCATGAAATTGTCGATCTAATGATTCACTATCAAGCAGTAG
TGGTACTGGAAAATCTGAATTTCGGTTTCAAAAGCAAACGTACAGGGATCGCTGAAAAAGCCGTTTATCAGCAGTTCGAGAAAATGCTGATAGACAAGCT
GAATTGCCTGGTTCTGAAAGATTATCCGGCAGAGAAGGTGGGCGGTGTGCTGAACCCGTACCAGCTGACTGATCAATTTACGAGCTTTGCAAAAATGGGA
ACGCAGAGCGGTTTCCTGTTCTATGTTCCGGCGCCATATACCAGCAAGATAGACCCGCTGACAGGTTTCGTAGATCCGTTTGTCTGGAAAACCATTAAAA
ATCATGAAAGTCGCAAACATTTTCTGGAGGGCTTTGATTTTCTGCACTATGACGTGAAAACCGGCGACTTCATTCTGCATTTTAAAATGAACCGTAATCT
GTCCTTTCAGCGCGGCCTGCCTGGCTTTATGCCGGCGTGGGACATTGTTTTTGAAAAGAATGAGACACAGTTTGATGCCAAAGGTACCCCCTTTATTGCG
GGGAAACGCATTGTGCCCGTTATAGAAAATCACCGCTTCACCGGACGGTATAGGGACTTGTACCCGGCAAATGAATTGATAGCGCTGCTGGAGGAGAAAG
GTATTGTCTTTCGGGATGGATCAAACATCCTGCCGAAGCTGCTGGAGAACGATGACAGCCACGCAATAGACACCATGGTAGCGCTGATCCGAAGCGTGCT
GCAGATGCGTAACAGTAATGCGGCTACGGGGGAAGACTACATTAATAGCCCGGTCCGTGATCTGAACGGCGTTTGTTTCGATAGCAGATTTCAAAATCCG
GAGTGGCCGATGGATGCCGATGCCAATGGAGCTTACCATATCGCTCTCAAAGGTCAGCTCCTACTGAACCATTTGAAAGAATCAAAAGATCTGAAACTGC
AGAACGGCATCTCGAATCAGGACTGGCTGGCCTACATTCAAGAACTGAGAAACCCAAAGAAAAAGAGAAAAGTAGGTGGTTACCCCTACGACGTGCCCGA
CTACGCCTAACACCCAGCTTTCTTGTACAAAGTGGTGATATCCCGCGGCCATGCTAGAGTCCGCAAAAATCACCAGTCTCTCTCTACAAATCTATCTCTC
TCTATTTTTCTCCAGAATAATGTGTGAGTAGTTCCCAGATAAGGGAATTAGGGTTCTTATAGGGTTTCGCTCATGTGTTGAGCATATAAGAAACCCTTAG
TATGTATTTGTATTTGTAAAATACTTCTATCAATAAAATTTCTAATTCCTAAAACCAAAATCCAGTGACCTGCAGGCATGCGACGTCGGGCCCAATTCGC
CCTATAGTGAGTCGTATTACAATTCACTGGCCGTCGTTTTACAACGTCGTGACTGGGAAAACCCTGGCGTTACCCAACTTAATCGCCTTGCAGCACATCC
CCCTTTCGCCAGCT

p2GW7-eLbCpf1-HA, *E.coli*-codon optimized LbCpf1.

Amp-R 35s promoter 5’ NLS eLbCpf1 3’ NLS HA Stop codon 35s terminator

GGCGTAATAGCGAAGAGGCCCGCACCGATCGCCCTTCCCAACAGTTGCGCAGCCTGAATGGCGAATGGACGCGCCCTGTAGCGGCGCATTAAGCGCGGCG
GGTGTGGTGGTTACGCGCAGCGTGACCGCTACACTTGCCAGCGCCCTAGCGCCCGCTCCTTTCGCTTTCTTCCCTTCCTTTCTCGCCACGTTCGCCGGCT
TTCCCCGTCAAGCTCTAAATCGGGGGCTCCCTTTAGGGTTCCGATTTAGTGCTTTACGGCACCTCGACCCCAAAAAACTTGATTAGGGTGATGGTTCACG
TAGTGGGCCATCGCCCTGATAGACGGTTTTTCGCCCTTTGACGTTGGAGTCCACGTTCTTTAATAGTGGACTCTTGTTCCAAACTGGAACAACACTCAAC
CCTATCTCGGTCTATTCTTTTGATTTATAAGGGATTTTGCCGATTTCGGCCTATTGGTTAAAAAATGAGCTGATTTAACAAAAATTTAACGCGAATTTTA
ACAAAATATTAACGCTTACAATTTCCTGATGCGGTATTTTCTCCTTACGCATCTGTGCGGTATTTCACACCGCATCAGGTGGCACTTTTCGGGGAAATGT
GCGCGGAACCCCTATTTGTTTATTTTTCTAAATACATTCAAATATGTATCCGCTCATGAGACAATAACCCTGATAAATGCTTCAATAATATTGAAAAAGG
AAGAGTATGAGTATTCAACATTTCCGTGTCGCCCTTATTCCCTTTTTTGCGGCATTTTGCCTTCCTGTTTTTGCTCACCCAGAAACGCTGGTGAAAGTAA
AAGATGCTGAAGATCAGTTGGGTGCACGAGTGGGTTACATCGAACTGGATCTCAACAGCGGTAAGATCCTTGAGAGTTTTCGCCCCGAAGAACGTTTTCC
AATGATGAGCACTTTTAAAGTTCTGCTATGTGGCGCGGTATTATCCCGTATTGACGCCGGGCAAGAGCAACTCGGTCGCCGCATACACTATTCTCAGAAT
GACTTGGTTGAGTACTCACCAGTCACAGAAAAGCATCTTACGGATGGCATGACAGTAAGAGAATTATGCAGTGCTGCCATAACCATGAGTGATAACACTG
CGGCCAACTTACTTCTGACAACGATCGGAGGACCGAAGGAGCTAACCGCTTTTTTGCACAACATGGGGGATCATGTAACTCGCCTTGATCGTTGGGAACC
GGAGCTGAATGAAGCCATACCAAACGACGAGCGTGACACCACGATGCCTGTAGCAATGGCAACAACGTTGCGCAAACTATTAACTGGCGAACTACTTACT
CTAGCTTCCCGGCAACAATTAATAGACTGGATGGAGGCGGATAAAGTTGCAGGACCACTTCTGCGCTCGGCCCTTCCGGCTGGCTGGTTTATTGCTGATA
AATCTGGAGCCGGTGAGCGTGGGTCTCGCGGTATCATTGCAGCACTGGGGCCAGATGGTAAGCCCTCCCGTATCGTAGTTATCTACACGACGGGGAGTCA
GGCAACTATGGATGAACGAAATAGACAGATCGCTGAGATAGGTGCCTCACTGATTAAGCATTGGTAACTGTCAGACCAAGTTTACTCATATATACTTTAG
ATTGATTTAAAACTTCATTTTTAATTTAAAAGGATCTAGGTGAAGATCCTTTTTGATAATCTCATGACCAAAATCCCTTAACGTGAGTTTTCGTTCCACT
GAGCGTCAGACCCCGTAGAAAAGATCAAAGGATCTTCTTGAGATCCTTTTTTTCTGCGCGTAATCTGCTGCTTGCAAACAAAAAAACCACCGCTACCAGC
GGTGGTTTGTTTGCCGGATCAAGAGCTACCAACTCTTTTTCCGAAGGTAACTGGCTTCAGCAGAGCGCAGATACCAAATACTGTTCTTCTAGTGTAGCCG
TAGTTAGGCCACCACTTCAAGAACTCTGTAGCACCGCCTACATACCTCGCTCTGCTAATCCTGTTACCAGTGGCTGCTGCCAGTGGCGATAAGTCGTGTC
TTACCGGGTTGGACTCAAGACGATAGTTACCGGATAAGGCGCAGCGGTCGGGCTGAACGGGGGGTTCGTGCACACAGCCCAGCTTGGAGCGAACGACCTA
CACCGAACTGAGATACCTACAGCGTGAGCTATGAGAAAGCGCCACGCTTCCCGAAGGGAGAAAGGCGGACAGGTATCCGGTAAGCGGCAGGGTCGGAACA
GGAGAGCGCACGAGGGAGCTTCCAGGGGGAAACGCCTGGTATCTTTATAGTCCTGTCGGGTTTCGCCACCTCTGACTTGAGCGTCGATTTTTGTGATGCT
CGTCAGGGGGGCGGAGCCTATGGAAAAACGCCAGCAACGCGGCCTTTTTACGGTTCCTGGCCTTTTGCTGGCCTTTTGCTCACATGTTCTTTCCTGCGTT
ATCCCCTGATTCTGTGGATAACCGTATTACCGCCTTTGAGTGAGCTGATACCGCTCGCCGCAGCCGAACGACCGAGCGCAGCGAGTCAGTGAGCGAGGAA
GCGGAAGAGCGCCCAATACGCAAACCGCCTCTCCCCGCGCGTTGGCCGATTCATTAATGCAGCTGGCACGACAGGTTTCCCGACTGGAAAGCGGGCAGTG
AGCGCAACGCAATTAATGTGAGTTAGCTCACTCATTAGGCACCCCAGGCTTTACACTTTATGCTTCCGGCTCGTATGTTGTGTGGAATTGTGAGCGGATA
ACAATTTCACACAGGAAACAGCTATGACCATGATTACGCCAAGCTATTTAGGTGACACTATAGAATACTCAAGCTATGCATCCAACGCGTTGGGAGCTCT
CCCATATGGTCGACTAGAGCCAAGCTGATCTCCTTTGCCCCGGAGATCACCATGGACGACTTTCTCTATCTCTACGATCTAGGAAGAAAGTTCGACGGAG
AAGGTGACGATACCATGTTCACCACCGATAATGAGAAGATTAGCCTCTTCAATTTCAGAAAGAATGCTGACCCACAGATGGTTAGAGAGGCCTACGCGGC
AGGTCTCATCAAGACGATCTACCCGAGTAATAATCTCCAGGAGATCAAATACCTTCCCAAGAAGGTTAAAGATGCAGTCAAAAGATTCAGGACTAACTGC
ATCAAGAACACAGAGAAAGATATATTTCTCAAGATCAGAAGTACTATTCCAGTATGGACGATTCAAGGCTTGCTTCATAAACCAAGGCAAGTAATAGAGA
TTGGAGTCTCTAAGAAAGTAGTTCCTACTGAATCAAAGGCCATGGAGTCAAAAATTCAGATCGAGGATCTAACAGAACTCGCCGTGAAGACTGGCGAACA
GTTCATACAGAGTCTTTTACGACTCAATGACAAGAAGAAAATCTTCGTCAACATGGTGGAGCACGACACTCTCGTCTACTCCAAGAATATCAAAGATACA
GTCTCAGAAGACCAAAGGGCTATTGAGACTTTTCAACAAAGGGTAATATCGGGAAACCTCCTCGGATTCCATTGCCCAGCTATCTGTCACTTCATCAAAA
GGACAGTAGAAAAGGAAGGTGGCACCTACAAATGCCATCATTGCGATAAAGGAAAGGCTATCGTTCAAGATGCCTCTGCCGACAGTGGTCCCAAAGATGG
ACCCCCACCCACGAGGAGCATCGTGGAAAAAGAAGACGTTCCAACCACGTCTTCAAAGCAAGTGGATTGATGTGATATCTCCACTGACGTAAGGGATGAC
GCACAATCCCACTATCCTTCGCAAGACCCTTCCTCTATATAAGGAAGTTCATTTCATTTGGAGAGGACTCCGGTATTTTTACAACAATACCACAACAAAA
CAAACAACAAACAACATTACAATTTACTATTCTAGTCGACCTGCAGGCGGCCGCACTAGTGATATCACAAGTTTGTACAAAAAAGCAGGCTTCATGGGTG
CTCCTCCAAAAAAGAAGAGAAAGGTAGGTGGTATGAGCAAACTGGAAAAATTTACGAATTGTTATAGCCTGTCCAAGACCCTGCGTTTCAAAGCCATCCC
CGTTGGCAAAACCCAGGAGAATATTGATAATAAACGTCTGCTGGTTGAGGATGAAAAAAGAGCAGAAGACTATAAGGGAGTCAAAAAACTGCTGGATCGG
TACTACCTGAGCTTTATAAATGACGTGCTGCATAGCATTAAACTGAAAAATCTGAATAACTATATTAGTCTGTTCCGCAAGAAAACCCGAACAGAGAAAG
AAAATAAAGAGCTGGAAAACCTGGAGATCAATCTGCGTAAAGAGATCGCAAAAGCTTTTAAAGGAAATGAAGGTTATAAAAGCCTGTTCAAAAAAGACAT
TATTGAAACCATCCTGCCGGAATTTCTGGATGATAAAGACGAGATAGCGCTCGTGAACAGCTTCAACGGGTTCACGACCGCCTTCACGGGCTTTTTCGAT
AACAGGGAAAATATGTTTTCAGAGGAAGCCAAAAGCACCTCGATAGCGTTCCGTTGCATTAATGAAAATTTGACAAGATATATCAGCAACATGGATATTT
TCGAGAAAGTTGATGCGATCTTTGACAAACATGAAGTGCAGGAGATTAAGGAAAAAATTCTGAACAGCGATTATGATGTTGAGGATTTTTTCGAGGGGA
ATTTTTTAACTTTGTACTGACACAGGAAGGTATAGATGTGTATAATGCTATTATCGGCGGGTTCGTTACCGAATCCGGCGAGAAAATTAAGGGTCTGAAT
GAGTACATCAATCTGTATAACCAAAAGACCAAACAGAAACTGCCAAAATTCAAACCGCTGTACAAGCAAGTCCTGAGCGATCGGGAAAGCTTGAGCTTTT
ACGGTGAAGGTTATACCAGCGACGAGGAGGTACTGGAGGTCTTTCGCAATACCCTGAACAAGAACAGCGAAATTTTCAGCTCCATTAAAAAGCTGGAGAA
ACTGTTTAAGAATTTTGACGAGTACAGCAGCGCAGGTATTTTTGTGAAGAACGGACCTGCCATAAGCACCATTAGCAAGGATATTTTTGGAGAGTGGAAT
GTTATCCGTGATAAATGGAACGCGGAATATGATGACATACACCTGAAAAAGAAGGCTGTGGTAACTGAGAAATATGAAGACGATCGCCGCAAAAGCTTTA
AAAAAATCGGCAGCTTTAGCCTGGAGCAGCTGCAGGAATATGCGGACGCCGACCTGAGCGTGGTCGAGAAACTGAAGGAAATTATTATCCAAAAAGTGGA
TGAGATTTACAAGGTATATGGTAGCAGCGAAAAACTGTTTGATGCGGACTTCGTTCTGGAAAAAAGCCTGAAAAAAAATGATGCTGTTGTTGCGATCATG
AAAGACCTGCTCGATAGCGTTAAGAGCTTTGAAAATTACATTAAAGCATTCTTTGGCGAGGGCAAAGAAACAAACAGAGACGAAAGCTTTTATGGCGACT

TCGTCCTGGCTTATGACATCCTGTTGAAGGTAGATCATATATATGATGCAATTCGTAATTACGTAACCCAAAAGCCGTACAGCAAAGATAAGTTCAAACT
GTATTTCCAGAACCCGCAGTTTATGGGTGGCTGGGACAAAGACAAGGAGACAGACTATCGCGCCACTATTCTGCGTTACGGCAGCAAGTACTATCTCGCC
ATCATGGACAAAAAATATGCAAAGTGTCTGCAGAAAATCGATAAAGACGACGTGAACGGAAATTACGAAAAGATTAATTATAAGCTGCTGCCAGGGCCCA
ACAAGATGTTACCGAAAGTATTTTTTTCCAAAAAATGGATGGCATACTATAACCCGAGCGAGGATATACAGAAGATTTACAAAAATGGGACCTTCAAAAA
GGGGGATATGTTCAATCTGAATGACTGCCACAAACTGATCGATTTTTTTAAAGATAGCATCAGCCGTTATCCTAAATGGTCAAACGCGTATGATTTTAAT
TTCTCCGAAACGGAGAAATATAAAGACATTGCTGGTTTCTATCGCGAAGTCGAAGAACAGGGTTATAAAGTTAGCTTTGAATCGGCCAGCAAGAAAGAGG
TTGATAAACTGGTGGAGGAGGGTAAGCTGTATATGTTTCAGATTTATAACAAAGACTTTAGCGACAAAAGCCACGGTACTCCTAATCTGCATACGATGTA
CTTTAAACTGCTGTTTGATGAGAATAACCACGGCCAAATCCGTCTCTCCGGTGGAGCAGAACTTTTTATGCGGCGTGCGAGCCTAAAAAAGGAAGAACTG
GTGGTGCATCCCGCCAACAGCCCGATTGCTAACAAAAATCCAGATAATCCTAAGAAGACCACCACACTGTCGTACGATGTCTATAAGGATAAACGTTTCT
CGGAAGACCAGTATGAATTGCATATACCGATAGCAATTAATAAATGCCCAAAAAACATTTTCAAAATCAACACTGAAGTTCGTGTGCTGCTGAAACATGA
TGATAATCCGTATGTGATCGGAATTGACCGTGGGGAGAGAAATCTGCTGTATATTGTAGTCGTTGATGGCAAGGGCAACATCGTTGAGCAGTATAGCCTG
AATGAAATAATTAATAATTTTAACGGTATACGTATTAAAACCGACTATCATAGCCTGCTGGATAAAAAGGAGAAAGAGCGTTTTGAGGCACGCCAAAATT
GGACGAGCATCGAAAACATCAAGGAACTGAAGGCAGGATATATCAGCCAAGTAGTCCATAAAATCTGTGAACTGGTGGAGAAGTACGACGCTGTCATTGC
CCTGGAAGACCTCAATAGCGGCTTTAAAAACAGCCGGGTGAAGGTGGAGAAACAGGTATACCAAAAGTTTGAAAAGATGCTCATTGATAAGCTGAACTAT
ATGGTTGATAAAAAGAGCAACCCGTGCGCCACTGGCGGTGCACTGAAAGGGTACCAAATTACCAATAAATTTGAAAGCTTTAAAAGCATGAGCACGCAGA
ATGGGTTTATTTTTTATATACCAGCATGGCTGACGAGCAAGATTGACCCCAGCACTGGTTTTGTCAATCTGCTGAAAACCAAATACACAAGCATTGCGGA
TAGCAAAAAATTTATTTCGAGCTTCGACCGTATTATGTATGTTCCGGAGGAAGATCTGTTTGAATTTGCCCTGGATTATAAAAACTTCAGCCGCACCGAT
GCAGATTATATCAAAAAATGGAAGCTGTACAGTTATGGTAATCGTATACGTATCTTCCGTAATCCGAAGAAAAACAATGTGTTCGATTGGGAAGAGGTCT
GTCTGACCAGCGCGTATAAAGAACTGTTCAACAAGTACGGAATAAATTATCAGCAAGGTGACATTCGCGCACTGCTGTGTGAACAGTCAGATAAAGCATT
TTATAGCAGCTTTATGGCGCTGATGAGCCTGATGCTCCAGATGCGCAACAGCATAACCGGTCGCACAGATGTTGACTTTCTGATCAGCCCTGTGAAGAAT
AGCGACGGCATCTTCTACGATTCCAGGAACTATGAAGCACAGGAAAACGCTATTCTGCCTAAAAATGCCGATGCCAACGGCGCCTATAATATTGCACGGA
AGGTTCTGTGGGCGATTGGACAGTTCAAGAAAGCGGAAGATGAGAAGCTGGATAAGGTAAAAATTGCTATTAGCAATAAGGAATGGCTGGAGTACGCACA
GACATCGGTTAAACACCCAAAGAAAAAGAGAAAAGTAGGTGGTTACCCCTACGACGTGCCCGACTACGCCTAACACCCAGCTTTCTTGTACAAAGTGGTG
ATATCCCGCGGCCATGCTAGAGTCCGCAAAAATCACCAGTCTCTCTCTACAAATCTATCTCTCTCTATTTTTCTCCAGAATAATGTGTGAGTAGTTCCCA
GATAAGGGAATTAGGGTTCTTATAGGGTTTCGCTCATGTGTTGAGCATATAAGAAACCCTTAGTATGTATTTGTATTTGTAAAATACTTCTATCAATAAA
ATTTCTAATTCCTAAAACCAAAATCCAGTGACCTGCAGGCATGCGACGTCGGGCCCAATTCGCCCTATAGTGAGTCGTATTACAATTCACTGGCCGTCGT
TTTACAACGTCGTGACTGGGAAAACCCTGGCGTTACCCAACTTAATCGCCTTGCAGCACATCCCCCTTTCGCCAGCT

p2GW7-AsCpf1-HA, plant-codon-optimized AsCpf1.

Amp-R 35s promoter 5’ NLS pAsCpf1 3’ NLS HA Stop codon 35s terminator

GGCGTAATAGCGAAGAGGCCCGCACCGATCGCCCTTCCCAACAGTTGCGCAGCCTGAATGGCGAATGGACGCGCCCTGTAGCGGCGCATTAAGCGCGGCG
GGTGTGGTGGTTACGCGCAGCGTGACCGCTACACTTGCCAGCGCCCTAGCGCCCGCTCCTTTCGCTTTCTTCCCTTCCTTTCTCGCCACGTTCGCCGGCT
TTCCCCGTCAAGCTCTAAATCGGGGGCTCCCTTTAGGGTTCCGATTTAGTGCTTTACGGCACCTCGACCCCAAAAAACTTGATTAGGGTGATGGTTCACG
TAGTGGGCCATCGCCCTGATAGACGGTTTTTCGCCCTTTGACGTTGGAGTCCACGTTCTTTAATAGTGGACTCTTGTTCCAAACTGGAACAACACTCAAC
CCTATCTCGGTCTATTCTTTTGATTTATAAGGGATTTTGCCGATTTCGGCCTATTGGTTAAAAAATGAGCTGATTTAACAAAAATTTAACGCGAATTTTA
ACAAAATATTAACGCTTACAATTTCCTGATGCGGTATTTTCTCCTTACGCATCTGTGCGGTATTTCACACCGCATCAGGTGGCACTTTTCGGGGAAATGT
GCGCGGAACCCCTATTTGTTTATTTTTCTAAATACATTCAAATATGTATCCGCTCATGAGACAATAACCCTGATAAATGCTTCAATAATATTGAAAAAGG
AAGAGTATGAGTATTCAACATTTCCGTGTCGCCCTTATTCCCTTTTTTGCGGCATTTTGCCTTCCTGTTTTTGCTCACCCAGAAACGCTGGTGAAAGTAA
AAGATGCTGAAGATCAGTTGGGTGCACGAGTGGGTTACATCGAACTGGATCTCAACAGCGGTAAGATCCTTGAGAGTTTTCGCCCCGAAGAACGTTTTCC
AATGATGAGCACTTTTAAAGTTCTGCTATGTGGCGCGGTATTATCCCGTATTGACGCCGGGCAAGAGCAACTCGGTCGCCGCATACACTATTCTCAGAAT
GACTTGGTTGAGTACTCACCAGTCACAGAAAAGCATCTTACGGATGGCATGACAGTAAGAGAATTATGCAGTGCTGCCATAACCATGAGTGATAACACTG
CGGCCAACTTACTTCTGACAACGATCGGAGGACCGAAGGAGCTAACCGCTTTTTTGCACAACATGGGGGATCATGTAACTCGCCTTGATCGTTGGGAACC
GGAGCTGAATGAAGCCATACCAAACGACGAGCGTGACACCACGATGCCTGTAGCAATGGCAACAACGTTGCGCAAACTATTAACTGGCGAACTACTTACT
CTAGCTTCCCGGCAACAATTAATAGACTGGATGGAGGCGGATAAAGTTGCAGGACCACTTCTGCGCTCGGCCCTTCCGGCTGGCTGGTTTATTGCTGATA
AATCTGGAGCCGGTGAGCGTGGGTCTCGCGGTATCATTGCAGCACTGGGGCCAGATGGTAAGCCCTCCCGTATCGTAGTTATCTACACGACGGGGAGTCA
GGCAACTATGGATGAACGAAATAGACAGATCGCTGAGATAGGTGCCTCACTGATTAAGCATTGGTAACTGTCAGACCAAGTTTACTCATATATACTTTAG
ATTGATTTAAAACTTCATTTTTAATTTAAAAGGATCTAGGTGAAGATCCTTTTTGATAATCTCATGACCAAAATCCCTTAACGTGAGTTTTCGTTCCACT
GAGCGTCAGACCCCGTAGAAAAGATCAAAGGATCTTCTTGAGATCCTTTTTTTCTGCGCGTAATCTGCTGCTTGCAAACAAAAAAACCACCGCTACCAGC
GGTGGTTTGTTTGCCGGATCAAGAGCTACCAACTCTTTTTCCGAAGGTAACTGGCTTCAGCAGAGCGCAGATACCAAATACTGTTCTTCTAGTGTAGCCG
TAGTTAGGCCACCACTTCAAGAACTCTGTAGCACCGCCTACATACCTCGCTCTGCTAATCCTGTTACCAGTGGCTGCTGCCAGTGGCGATAAGTCGTGTC
TTACCGGGTTGGACTCAAGACGATAGTTACCGGATAAGGCGCAGCGGTCGGGCTGAACGGGGGGTTCGTGCACACAGCCCAGCTTGGAGCGAACGACCTA
CACCGAACTGAGATACCTACAGCGTGAGCTATGAGAAAGCGCCACGCTTCCCGAAGGGAGAAAGGCGGACAGGTATCCGGTAAGCGGCAGGGTCGGAACA
GGAGAGCGCACGAGGGAGCTTCCAGGGGGAAACGCCTGGTATCTTTATAGTCCTGTCGGGTTTCGCCACCTCTGACTTGAGCGTCGATTTTTGTGATGCT
CGTCAGGGGGGCGGAGCCTATGGAAAAACGCCAGCAACGCGGCCTTTTTACGGTTCCTGGCCTTTTGCTGGCCTTTTGCTCACATGTTCTTTCCTGCGTT
ATCCCCTGATTCTGTGGATAACCGTATTACCGCCTTTGAGTGAGCTGATACCGCTCGCCGCAGCCGAACGACCGAGCGCAGCGAGTCAGTGAGCGAGGAA
GCGGAAGAGCGCCCAATACGCAAACCGCCTCTCCCCGCGCGTTGGCCGATTCATTAATGCAGCTGGCACGACAGGTTTCCCGACTGGAAAGCGGGCAGTG
AGCGCAACGCAATTAATGTGAGTTAGCTCACTCATTAGGCACCCCAGGCTTTACACTTTATGCTTCCGGCTCGTATGTTGTGTGGAATTGTGAGCGGATA
ACAATTTCACACAGGAAACAGCTATGACCATGATTACGCCAAGCTATTTAGGTGACACTATAGAATACTCAAGCTATGCATCCAACGCGTTGGGAGCTCT
CCCATATGGTCGACTAGAGCCAAGCTGATCTCCTTTGCCCCGGAGATCACCATGGACGACTTTCTCTATCTCTACGATCTAGGAAGAAAGTTCGACGGAG
AAGGTGACGATACCATGTTCACCACCGATAATGAGAAGATTAGCCTCTTCAATTTCAGAAAGAATGCTGACCCACAGATGGTTAGAGAGGCCTACGCGGC
AGGTCTCATCAAGACGATCTACCCGAGTAATAATCTCCAGGAGATCAAATACCTTCCCAAGAAGGTTAAAGATGCAGTCAAAAGATTCAGGACTAACTGC
ATCAAGAACACAGAGAAAGATATATTTCTCAAGATCAGAAGTACTATTCCAGTATGGACGATTCAAGGCTTGCTTCATAAACCAAGGCAAGTAATAGAGA
TTGGAGTCTCTAAGAAAGTAGTTCCTACTGAATCAAAGGCCATGGAGTCAAAAATTCAGATCGAGGATCTAACAGAACTCGCCGTGAAGACTGGCGAACA
GTTCATACAGAGTCTTTTACGACTCAATGACAAGAAGAAAATCTTCGTCAACATGGTGGAGCACGACACTCTCGTCTACTCCAAGAATATCAAAGATACA
GTCTCAGAAGACCAAAGGGCTATTGAGACTTTTCAACAAAGGGTAATATCGGGAAACCTCCTCGGATTCCATTGCCCAGCTATCTGTCACTTCATCAAAA
GGACAGTAGAAAAGGAAGGTGGCACCTACAAATGCCATCATTGCGATAAAGGAAAGGCTATCGTTCAAGATGCCTCTGCCGACAGTGGTCCCAAAGATGG
ACCCCCACCCACGAGGAGCATCGTGGAAAAAGAAGACGTTCCAACCACGTCTTCAAAGCAAGTGGATTGATGTGATATCTCCACTGACGTAAGGGATGAC
GCACAATCCCACTATCCTTCGCAAGACCCTTCCTCTATATAAGGAAGTTCATTTCATTTGGAGAGGACTCCGGTATTTTTACAACAATACCACAACAAAA
CAAACAACAAACAACATTACAATTTACTATTCTAGTCGACCTGCAGGCGGCCGCACTAGTGATATCACAAGTTTGTACAAAAAAGCAGGCTTCATGGCCC
CAAAGAAGAAGCGGAAGGTCGGTATCCACGGAGTCCCAGCAGCCATGACTCAGTTTGAGGGCTTCACCAATCTCTACCAGGTTAGTAAGACGCTAAGATT
TGAGCTAATCCCGCAAGGAAAGACTCTAAAACACATTCAAGAACAAGGGTTCATAGAGGAGGATAAGGCGAGAAATGACCATTATAAAGAACTGAAGCCT
ATAATTGACCGTATTTATAAAACATACGCGGATCAGTGCTTACAACTGGTTCAACTGGATTGGGAGAACCTGTCCGCGGCTATTGATAGCTATAGAAAAG
AGAAGACTGAGGAAACAAGGAATGCTCTGATTGAGGAGCAAGCTACCTACCGGAACGCGATCCATGATTACTTCATCGGTAGAACTGACAACCTGACTGA

TGCAATCAACAAGAGACACGCAGAGATTTACAAAGGTTTGTTCAAGGCAGAGTTATTCAACGGCAAGGTTCTCAAACAGCTGGGTACGGTCACCACAACC
GAGCATGAGAACGCTCTGCTGAGAAGCTTTGATAAGTTTACAACATATTTCTCAGGTTTCTATGAGAATCGTAAGAATGTATTTAGCGCTGAAGATATAT
CAACAGCTATTCCACATAGGATTGTGCAGGATAACTTTCCAAAGTTCAAAGAAAATTGTCATATTTTTACACGTCTGATCACCGCCGTACCATCGCTGCG
AGAGCATTTCGAGAACGTTAAGAAAGCCATAGGAATTTTTGTCAGTACATCAATTGAGGAAGTGTTTTCGTTCCCATTCTATAACCAGCTGCTGACGCAG
ACCCAGATTGATCTCTACAATCAGTTGCTCGGTGGAATAAGCCGCGAGGCAGGTACTGAAAAGATAAAGGGACTCAACGAGGTACTGAATCTGGCAATTC
AAAAAAATGATGAAACGGCTCATATAATTGCTAGCCTGCCACATAGATTCATACCCCTGTTTAAGCAAATCCTGAGCGATAGAAATACACTGAGCTTTAT
CTTGGAGGAGTTTAAGTCGGACGAGGAGGTTATCCAATCCTTTTGCAAGTACAAAACTCTCCTGCGGAACGAGAATGTGCTGGAGACAGCTGAAGCACTG
TTTAATGAACTGAACTCAATCGATCTCACTCATATCTTTATATCCCATAAGAAACTGGAGACCATAAGCAGCGCTCTGTGTGATCACTGGGATACGCTGA
GGAACGCCCTGTATGAAAGACGTATTAGCGAGCTGACCGGGAAAATCACGAAGTCCGCTAAGGAAAAAGTTCAGAGGAGTCTAAAACACGAGGACATCAA
CCTGCAAGAAATTATTAGCGCTGCAGGAAAGGAGCTGAGCGAGGCTTTCAAGCAAAAGACCAGCGAAATCTTGAGCCATGCCCATGCTGCGCTGGATCAA
CCTCTGCCTACAACCCTGAAGAAGCAGGAGGAGAAGGAAATTCTGAAATCTCAGCTGGACTCACTGCTGGGCCTGTATCACCTTCTTGATTGGTTTGCTG
TCGATGAGAGCAACGAGGTTGATCCTGAGTTCTCCGCTCGACTGACCGGAATCAAGCTGGAGATGGAGCCTAGTCTGAGTTTCTATAATAAAGCTCGAAA
CTACGCAACAAAAAAACCGTACTCTGTGGAAAAATTCAAACTGAATTTTCAAATGCCAACGCTCGCAAGTGGATGGGACGTTAATAAGGAAAAGAATAAC
GGGGCCATTCTGTTCGTGAAAAATGGACTCTATTATCTGGGTATCATGCCGAAACAAAAGGGGAGGTACAAGGCCCTTTCATTCGAGCCTACTGAGAAAA
CCTCAGAGGGTTTCGACAAGATGTACTACGATTATTTCCTGACGCTGCAAAAATGATACCCAAGTGTTCAACTCAACTGAAAGCTGTTACAGCGCACTT
TCAGACTCACACCACCCCTATCCTCCTCTCGAACAATTTTATAGAGCCACTGGAAATTACTAAAGAGATTTATGATCTGAATAATCCGGAAAAGGAGCCC
AAGAAGTTCCAGACAGCTTACGCAAAAAAAACCGGGGACCAGAAGGGTTATCGTGAAGCACTATGCAAGTGGATTGACTTTACCAGGGACTTTCTGTCAA
AATATACAAAAACTACATCGATTGATTTGTCAAGCCTAAGGCCAAGCTCTCAATACAAGGATCTGGGTGAATACTACGCCGAACTGAACCCGCTTCTGTA
CCATATCTCCTTCCAGCGAATAGCTGAAAAAGAAATCATGGATGCTGTTGAAACTGGGAAACTGTATCTGTTTCAAATCTATAATAAGGATTTTGCTAAA
GGGCATCACGGTAAGCCTAACTTGCATACCCTGTACTGGACTGGTCTTTTCAGTCCTGAGAATCTCGCTAAGACATCTATCAAGCTGAATGGACAGGCAG
AACTGTTTTACAGACCCAAAAGCCGAATGAAAAGGATGGCACACAGACTGGGAGAGAAGATGCTGAACAAGAAGCTCAAGGATCAAAAAACTCCGATCCC
AGATACTTTGTATCAAGAGCTGTATGATTACGTTAACCACAGACTGAGCCATGATTTGAGTGACGAAGCTAGGGCACTACTGCCGAACGTGATTACGAAG
GAGGTCTCGCATGAAATTATTAAAGATCGGCGCTTCACATCTGATAAGTTTTTCTTCCATGTACCTATTACACTGAACTACCAAGCCGCAAATTCACCTT
CCAAATTTAATCAAAGAGTGAATGCGTATCTAAAAGAACATCCAGAGACTCCAATTATTGGAATAGATCGAGGAGAGAGAAATCTCATTTACATCACGGT
CATCGATAGCACAGGTAAGATCCTGGAACAGCGTTCTCTTAATACAATTCAGCAATTTGATTACCAGAAGAAGCTAGACAACAGAGAAAAGGAGAGGGTA
GCTGCACGGCAAGCTTGGAGTGTTGTGGGTACTATCAAGGATCTGAAGCAGGGATATTTATCCCAAGTTATACATGAAATAGTCGACCTCATGATACACT
ACCAAGCTGTGGTGGTACTGGAGAATCTGAATTTCGGTTTCAAAAGCAAACGTACTGGGATCGCTGAAAAAGCCGTTTATCAGCAGTTTGAGAAAATGCT
GATAGACAAGCTTAATTGTCTGGTTCTGAAAGATTATCCAGCAGAGAAGGTTGGAGGTGTGCTGAACCCTTACCAACTGACTGATCAATTCACGTCTTTT
GCAAAAATGGGCACGCAGAGTGGTTTCCTGTTTTATGTTCCTGCACCATATACCTCTAAAATAGACCCTCTGACAGGATTCGTAGATCCTTTTGTCTGGA
AAACCATTAAGAATCACGAAAGTAGAAAACATTTCCTGGAGGGCTTTGACTTCCTGCACTACGATGTTAAGACTGGCGATTTCATTCTGCATTTCAAGAT
GAACCGTAATCTCTCCTTTCAACGCGGATTGCCTGGCTTTATGCCGGCGTGGGATATTGTTTTTGAAAAGAATGAGACACAATTTGATGCCAAGGGTACC
CCATTCATCGCGGGAAAAAGAATTGTGCCCGTTATCGAAAACCACAGATTCACTGGACGGTATAGGGACTTATACCCTGCGAACGAATTGATAGCTCTCC
TGGAGGAGAAAGGTATTGTCTTTAGGGACGGATCAAACATCCTCCCTAAACTGCTCGAGAACGATGACTCACACGCTATAGATACCATGGTTGCTTTGAT
CCGAAGCGTGCTCCAGATGCGTAACTCTAATGCGGCTACGGGGGAGGACTACATAAATAGCCCAGTCCGTGATCTGAACGGCGTTTGTTTCGACTCAAGA
TTCCAAAATCCTGAATGGCCAATGGATGCCGATGCCAATGGAGCTTACCATATCGCTCTCAAAGGTCAACTCCTACTAAACCATTTGAAGGAGTCAAAAG
ATCTGAAGCTCCAGAACGGAATCTCGAACCAGGATTGGTTGGCTTATATTCAAGAGCTGAGAAACAAAAGGCCGGCGGCCACGAAAAAGGCCGGCCAGGC
AAAAAAGAAAAAGGGATCCTACCCATACGATGTTCCAGATTACGCTTATCCCTACGACGTGCCTGATTATGCATACCCATATGATGTCCCCGACTATGCC
TAACACCCAGCTTTCTTGTACAAAGTGGTGATATCCCGCGGCCATGCTAGAGTCCGCAAAAATCACCAGTCTCTCTCTACAAATCTATCTCTCTCTATTT
TTCTCCAGAATAATGTGTGAGTAGTTCCCAGATAAGGGAATTAGGGTTCTTATAGGGTTTCGCTCATGTGTTGAGCATATAAGAAACCCTTAGTATGTAT
TTGTATTTGTAAAATACTTCTATCAATAAAATTTCTAATTCCTAAAACCAAAATCCAGTGACCTGCAGGCATGCGACGTCGGGCCCAATTCGCCCTATAG
TGAGTCGTATTACAATTCACTGGCCGTCGTTTTACAACGTCGTGACTGGGAAAACCCTGGCGTTACCCAACTTAATCGCCTTGCAGCACATCCCCCTTTC
GCCAGCT

p2GW7-LbCpf1-HA, plant-codon-optimized LbCpf1.

Amp-R35s promoter5' NLS pLbCpf13' NLS HA Stop codon35s terminator

GGCGTAATAGCGAAGAGGCCCGCACCGATCGCCCTTCCCAACAGTTGCGCAGCCTGAATGGCGAATGGACGCGCCCTGTAGCGGCGCATTAAGCGCGGCG
GGTGTGGTGGTTACGCGCAGCGTGACCGCTACACTTGCCAGCGCCCTAGCGCCCGCTCCTTTCGCTTTCTTCCCTTCCTTTCTCGCCACGTTCGCCGGCT
TTCCCCGTCAAGCTCTAAATCGGGGGCTCCCTTTAGGGTTCCGATTTAGTGCTTTACGGCACCTCGACCCCAAAAAACTTGATTAGGGTGATGGTTCACG
TAGTGGGCCATCGCCCTGATAGACGGTTTTTCGCCCTTTGACGTTGGAGTCCACGTTCTTTAATAGTGGACTCTTGTTCCAAACTGGAACAACACTCAAC
CCTATCTCGGTCTATTCTTTTGATTTATAAGGGATTTTGCCGATTTCGGCCTATTGGTTAAAAAATGAGCTGATTTAACAAAAATTTAACGCGAATTTTA
ACAAAATATTAACGCTTACAATTTCCTGATGCGGTATTTTCTCCTTACGCATCTGTGCGGTATTTCACACCGCATCAGGTGGCACTTTTCGGGGAAATGT
GCGCGGAACCCCTATTTGTTTATTTTTCTAAATACATTCAAATATGTATCCGCTCATGAGACAATAACCCTGATAAATGCTTCAATAATATTGAAAAAGG
AAGAGTATGAGTATTCAACATTTCCGTGTCGCCCTTATTCCCTTTTTTGCGGCATTTTGCCTTCCTGTTTTTGCTCACCCAGAAACGCTGGTGAAAGTAA
AAGATGCTGAAGATCAGTTGGGTGCACGAGTGGGTTACATCGAACTGGATCTCAACAGCGGTAAGATCCTTGAGAGTTTTCGCCCCGAAGAACGTTTTCC
AATGATGAGCACTTTTAAAGTTCTGCTATGTGGCGCGGTATTATCCCGTATTGACGCCGGGCAAGAGCAACTCGGTCGCCGCATACACTATTCTCAGAAT
GACTTGGTTGAGTACTCACCAGTCACAGAAAAGCATCTTACGGATGGCATGACAGTAAGAGAATTATGCAGTGCTGCCATAACCATGAGTGATAACACTG
CGGCCAACTTACTTCTGACAACGATCGGAGGACCGAAGGAGCTAACCGCTTTTTTGCACAACATGGGGGATCATGTAACTCGCCTTGATCGTTGGGAACC
GGAGCTGAATGAAGCCATACCAAACGACGAGCGTGACACCACGATGCCTGTAGCAATGGCAACAACGTTGCGCAAACTATTAACTGGCGAACTACTTACT
CTAGCTTCCCGGCAACAATTAATAGACTGGATGGAGGCGGATAAAGTTGCAGGACCACTTCTGCGCTCGGCCCTTCCGGCTGGCTGGTTTATTGCTGATA
AATCTGGAGCCGGTGAGCGTGGGTCTCGCGGTATCATTGCAGCACTGGGGCCAGATGGTAAGCCCTCCCGTATCGTAGTTATCTACACGACGGGGAGTCA
GGCAACTATGGATGAACGAAATAGACAGATCGCTGAGATAGGTGCCTCACTGATTAAGCATTGGTAACTGTCAGACCAAGTTTACTCATATATACTTTAG
ATTGATTTAAAACTTCATTTTTAATTTAAAAGGATCTAGGTGAAGATCCTTTTTGATAATCTCATGACCAAAATCCCTTAACGTGAGTTTTCGTTCCACT
GAGCGTCAGACCCCGTAGAAAAGATCAAAGGATCTTCTTGAGATCCTTTTTTTCTGCGCGTAATCTGCTGCTTGCAAACAAAAAAACCACCGCTACCAGC
GGTGGTTTGTTTGCCGGATCAAGAGCTACCAACTCTTTTTCCGAAGGTAACTGGCTTCAGCAGAGCGCAGATACCAAATACTGTTCTTCTAGTGTAGCCG
TAGTTAGGCCACCACTTCAAGAACTCTGTAGCACCGCCTACATACCTCGCTCTGCTAATCCTGTTACCAGTGGCTGCTGCCAGTGGCGATAAGTCGTGTC
TTACCGGGTTGGACTCAAGACGATAGTTACCGGATAAGGCGCAGCGGTCGGGCTGAACGGGGGGTTCGTGCACACAGCCCAGCTTGGAGCGAACGACCTA
CACCGAACTGAGATACCTACAGCGTGAGCTATGAGAAAGCGCCACGCTTCCCGAAGGGAGAAAGGCGGACAGGTATCCGGTAAGCGGCAGGGTCGGAACA
GGAGAGCGCACGAGGGAGCTTCCAGGGGGAAACGCCTGGTATCTTTATAGTCCTGTCGGGTTTCGCCACCTCTGACTTGAGCGTCGATTTTTGTGATGCT
CGTCAGGGGGGCGGAGCCTATGGAAAAACGCCAGCAACGCGGCCTTTTTACGGTTCCTGGCCTTTTGCTGGCCTTTTGCTCACATGTTCTTTCCTGCGTT
ATCCCCTGATTCTGTGGATAACCGTATTACCGCCTTTGAGTGAGCTGATACCGCTCGCCGCAGCCGAACGACCGAGCGCAGCGAGTCAGTGAGCGAGGAA
GCGGAAGAGCGCCCAATACGCAAACCGCCTCTCCCCGCGCGTTGGCCGATTCATTAATGCAGCTGGCACGACAGGTTTCCCGACTGGAAAGCGGGCAGTG
AGCGCAACGCAATTAATGTGAGTTAGCTCACTCATTAGGCACCCCAGGCTTTACACTTTATGCTTCCGGCTCGTATGTTGTGTGGAATTGTGAGCGGATA
ACAATTTCACACAGGAAACAGCTATGACCATGATTACGCCAAGCTATTTAGGTGACACTATAGAATACTCAAGCTATGCATCCAACGCGTTGGGAGCTCT

CCCATATGGTCGACTAGAGCCAAGCTGATCTCCTTTGCCCCGGAGATCACCATGGACGACTTTCTCTATCTCTACGATCTAGGAAGAAAGTTCGACGGAG
AAGGTGACGATACCATGTTCACCACCGATAATGAGAAGATTAGCCTCTTCAATTTCAGAAAGAATGCTGACCCACAGATGGTTAGAGAGGCCTACGCGGC
AGGTCTCATCAAGACGATCTACCCGAGTAATAATCTCCAGGAGATCAAATACCTTCCCAAGAAGGTTAAAGATGCAGTCAAAAGATTCAGGACTAACTGC
ATCAAGAACACAGAGAAAGATATATTTCTCAAGATCAGAAGTACTATTCCAGTATGGACGATTCAAGGCTTGCTTCATAAACCAAGGCAAGTAATAGAGA
TTGGAGTCTCTAAGAAAGTAGTTCCTACTGAATCAAAGGCCATGGAGTCAAAAATTCAGATCGAGGATCTAACAGAACTCGCCGTGAAGACTGGCGAACA
GTTCATACAGAGTCTTTTACGACTCAATGACAAGAAGAAAATCTTCGTCAACATGGTGGAGCACGACACTCTCGTCTACTCCAAGAATATCAAAGATACA
GTCTCAGAAGACCAAAGGGCTATTGAGACTTTTCAACAAAGGGTAATATCGGGAAACCTCCTCGGATTCCATTGCCCAGCTATCTGTCACTTCATCAAAA
GGACAGTAGAAAAGGAAGGTGGCACCTACAAATGCCATCATTGCGATAAAGGAAAGGCTATCGTTCAAGATGCCTCTGCCGACAGTGGTCCCAAAGATGG
ACCCCCACCCACGAGGAGCATCGTGGAAAAAGAAGACGTTCCAACCACGTCTTCAAAGCAAGTGGATTGATGTGATATCTCCACTGACGTAAGGGATGAC
GCACAATCCCACTATCCTTCGCAAGACCCTTCCTCTATATAAGGAAGTTCATTTCATTTGGAGAGGACTCCGGTATTTTTACAACAATACCACAACAAAA
CAAACAACAAACAACATTACAATTTACTATTCTAGTCGACCTGCAGGCGGCCGCACTAGTGATATCACAAGTTTGTACAAAAAAGCAGGCTTCATGGCCC
CAAAGAAGAAGCGGAAGGTCGGGATCCACGGAGTCCCAGCAGCCATGAGTAAGCTGGAGAAATTTACGAATTGTTATAGCCTGTCCAAGACACTGAGGTT
CAAGGCTATCCCCGTTGGAAAAACTCAAGAGAATATCGATAACAAGCGTCTGCTGGTTGAGGATGAGAAGAGAGCGGAAGACTATAAGGGAGTCAAAAAA
CTTCTGGATAGATACTATCTGTCTTTTATAAACGACGTGCTGCATTCCATTAAATTGAAGAATCTGAACAACTATATTTCCCTGTTCCGAAAGAAGACGA
GAACAGAGAAAGAAAATAAGGAGCTGGAAAACCTGGAGATCAACCTGAGGAAGGAGATTGCAAAGGCTTTCAAGGGAAATGAAGGTTATAAGAGCCTGTT
CAAAAAAGATATTATTGAAACTATACTGCCTGAATTTCTCGATGATAAGGATGAGATCGCGCTCGTGAACAGCTTTAACGGATTCACGACTGCCTTTACT
GGATTTTTCGATAATAGGGAGAACATGTTCTCAGAGGAAGCCAAGAGCACCTCGATCGCTTTTCGTTGCATAAATGAGAATTTGACAAGATACATCAGCA
ACATGGACATTTTCGAGAAGGTTGATGCGATCTTTGATAAGCATGAGGTTCAGGAGATTAAGGAGAAGATTCTGAACTCTGATTATGATGTTGAGGATTT
CTTCGAAGGAGAATTTTTAATTTCGTTTTGACACAGGAAGGTATAGATGTGTATAACGCTATTATCGGTGGGTTCGTTACCGAATCAGGGGAGAAGATA
AAGGGTCTGAATGAGTACATCAATCTGTATAACCAGAAGACAAAGCAGAAACTGCCAAAATTCAAGCCTCTCTATAAGCAAGTCCTGAGCGATCGGGAGT
CGCTTTCGTTCTACGGTGAAGGTTATACCAGCGATGAGGAGGTACTGGAGGTCTTTAGAAACACTCTGAACAAGAATAGCGAAATTTTCTCTTCCATTAA
GAAGCTGGAGAAGCTGTTCAAGAATTTTGATGAGTACTCGAGCGCAGGTATTTTCGTGAAGAACGGACCTGCTATAAGCACTATTAGCAAGGATATTTTT
GGAGAGTGGAATGTTATTCGGGATAAGTGGAATGCAGAGTACGACGATATACACCTGAAGAAGAAGGCTGTGGTAACTGAGAAGTATGAGGACGATAGAC
GCAAAAGCTTCAAGAAGATCGGTTCCTTTAGCCTGGAGCAACTGCAGGAGTATGCGGACGCAGATCTGAGCGTGGTCGAGAAACTGAAGGAGATCATTAT
CCAAAAGGTGGATGAGATTTACAAGGTATACGGTAGCTCAGAAAAGCTCTTTGATGCAGATTTCGTTCTCGAAAAGAGCCTGAAGAAGAATGATGCTGTT
GTTGCTATAATGAAGGACCTGCTCGATAGCGTTAAGAGCTTTGAGAATTACATCAAGGCATTCTTTGGCGAGGGAAAGGAAACAAACAGAGACGAAAGCT
TCTATGGCGACTTTGTGCTAGCTTATGACATCCTGCTGAAGGTAGACCATATATATGATGCAATTCGTAATTACGTTACCCAAAAGCCGTACAGCAAGGA
TAAGTTCAAACTCTATTTTCAAAACCCGCAATTTATGGGTGGCTGGGATAAGGACAAGGAAACAGATTATAGGGCCACTATCCTGCGATATGGCAGCAAG
TACTACCTCGCTATCATGGATAAGAAGTATGCTAAGTGTCTCCAGAAGATAGATAAGGACGACGTGAACGGAAATTACGAGAAGATTAATTACAAGCTAC
TGCCTGGGCCTAACAAGATGCTGCCAAAAGTCTTCTTTTCAAAGAAATGGATGGCTTACTACAACCCATCAGAGGATATCCAAAAGATATACAAAAATGG
GACCTTTAAAAAGGGAGACATGTTTAACCTGAATGACTGCCATAAGCTGATCGATTTTTTTAAGGATAGCATCAGCAGATATCCTAAGTGGTCAAACGCT
TATGATTTTAATTTCTCCGAAACAGAGAAGTATAAGGACATTGCTGGTTTCTATAGGGAAGTCGAAGAACAAGGTTATAAGGTTAGCTTTGAATCGGCTT
CAAAGAAGGAAGTTGATAAACTAGTGGAAGAAGGTAAGCTCTATATGTTCCAAATTTACAACAAGGATTTTTCCGACAAGTCTCACGGAACTCCTAACCT
TCATACGATGTATTTTAAGCTGCTGTTTGATGAGAACAATCACGGCCAAATACGACTCTCCGGTGGAGCAGAACTTTTTATGCGGAGAGCGAGCCTCAAG
AAAGAAGAACTTGTGGTTCATCCCGCCAACAGCCCGATTGCTAACAAGAATCCAGATAACCCTAAGAAGACTACAACACTGTCGTACGATGTTTATAAGG
ATAAGCGTTTCTCGGAAGACCAGTATGAGTTGCACATACCAATAGCAATCAATAAGTGCCCAAAGAACATTTTCAAAATCAACACCGAGGTTCGTGTTCT
GCTGAAGCATGACGATAACCCGTACGTGATAGGAATTGACAGGGGGGAGAGAAACCTGCTGTATATCGTAGTCGTTGATGGAAAGGGCAACATCGTTGAG
CAGTACAGCTTGAATGAAATCATTAACAACTTTAACGGTATACGTATCAAGACTGACTACCATAGCCTGCTGGATAAGAAGGAGAAGGAGAGATTCGAGG
CAAGGCAAAACTGGACGAGCATCGAAAACATCAAGGAACTAAAGGCCGGTTATATCAGCCAAGTAGTCCATAAGATTTGTGAGCTGGTGGAGAAGTATGA
CGCTGTCATCGCCTTGGAGGATCTGAATTCTGGCTTCAAAAATAGCCGGGTGAAAGTGGAGAAGCAGGTATACCAAAAGTTTGAGAAGATGCTCATCGAT
AAGCTAAACTACATGGTTGATAAAAAGAGCAATCCGTGCGCCACAGGAGGAGCTCTCAAAGGGTACCAAATAACCAACAAGTTCGAAAGCTTCAAGTCAA
TGAGCACACAAAACGGGTTTATTTTTTATATACCAGCATGGCTGACGAGCAAGATTGATCCCAGCACTGGTTTTGTCAACCTGCTGAAGACCAAGTATAC
TAGCATTGCTGATTCTAAAAAGTTTATTTCGAGCTTCGACCGTATTATGTACGTTCCAGAGGAGGATCTGTTCGAATTCGCGCTGGATTACAAGAACTTC
AGCAGAACCGATGCTGATTATATTAAGAAGTGGAAGCTGTACTCTTACGGTAATAGAATAAGGATCTTCAGAAACCCTAAGAAAAACAACGTGTTCGATT
GGGAAGAGGTCTGTCTCACTAGCGCGTATAAGGAGCTGTTCAATAAGTACGGAATAAATTACCAGCAAGGGGACATCCGCGCTCTGCTGTGTGAACAGAG
TGACAAAGCATTTTATAGCAGTTTCATGGCGCTGATGAGCCTGATGCTGCAGATGCGAAACAGCATTACTGGTAGAACAGACGTTGACTTTCTCATCAGC
CCTGTGAAGAACAGCGACGGAATCTTTTACGATTCCAGGAACTACGAGGCACAGGAAAACGCTATTCTGCCTAAAAACGCTGATGCAAACGGAGCCTATA
ACATTGCACGAAAAGTTCTGTGGGCTATTGGACAGTTCAAGAAGGCTGAAGATGAGAAGCTAGATAAGGTAAAGATAGCTATTAGCAATAAGGAATGGCT
GGAGTACGCACAAACATCCGTTAAGCACAAAAGGCCGGCGGCCACGAAAAAGGCCGGCCAGGCAAAAAAGAAAAAGGGATCCTACCCATACGATGTTCCA
GATTACGCTTATCCCTACGACGTGCCTGATTATGCATACCCATATGATGTCCCCGACTATGCCTAACACCCAGCTTTCTTGTACAAAGTGGTGATATCCC
GCGGCCATGCTAGAGTCCGCAAAAATCACCAGTCTCTCTCTACAAATCTATCTCTCTCTATTTTTCTCCAGAATAATGTGTGAGTAGTTCCCAGATAAGG
GAATTAGGGTTCTTATAGGGTTTCGCTCATGTGTTGAGCATATAAGAAACCCTTAGTATGTATTTGTATTTGTAAAATACTTCTATCAATAAAATTTCTA
ATTCCTAAAACCAAAATCCAGTGACCTGCAGGCATGCGACGTCGGGCCCAATTCGCCCTATAGTGAGTCGTATTACAATTCACTGGCCGTCGTTTTACAA
CGTCGTGACTGGGAAAACCCTGGCGTTACCCAACTTAATCGCCTTGCAGCACATCCCCCTTTCGCCAGCT
